# Supplementary material for: Deep-ultraviolet electroluminescence and photocurrent generation in graphene/hBN/graphene heterostructures
Source: Nat Commun. 2021 Dec 8;12:7134. doi: 10.1038/s41467-021-27524-w (PMC8654827; doi:10.1038/s41467-021-27524-w)
Supplement: Supplementary file 1 — Supplementary Information [file 41467_2021_27524_MOESM1_ESM.pdf]

Supplementary Information for

Deep-ultraviolet electroluminescence and photocurrent generation in graphene/hBN/graphene heterostructures

Su-Beom Song[1,2]*, Sangho Yoon[1,2]*, So Young Kim[1,2,3], Sera Yang[1,2], Seung-Young Seo[1,2], Soonyoung Cha[1,2], Hyeon-Woo Jeong[3], Kenji Watanabe[4], Takashi Taniguchi[5], Gil-Ho Lee[3], Jun Sung Kim[2,3], Moon-Ho Jo[1,2], Jonghwan Kim[1,2,3]†

[1]Department of Materials Science and Engineering, Pohang University of Science and Technology, Pohang, Republic of Korea

[2]Center for Artificial Low Dimensional Electronic Systems, Institute for Basic Science (IBS), Pohang, Republic of Korea

[3]Department of Physics, Pohang University of Science and Technology, Pohang, Republic of Korea

[4]Research Center for Functional Materials, National Institute for Materials Science, Tsukuba, Ibaraki, Japan

[5]International Center for Materials Nanoarchitectonics, National Institute for Materials Science, Tsukuba, Ibaraki, Japan

* These authors contributed equally to this work

† To whom correspondence should be addressed: jonghwankim@postech.ac.kr (J.K.)

Supplementary Note 1. Electroluminescence from device structures with and without hBN encapsulation at a temperature of 10 K

Supplementary Figure 1 shows electroluminescence spectra from representative devices without hBN encapsulation. The device structure of Gr/hBN/Gr alone (Supplementary Figure 1a and 1b) typically shows broad electroluminescence spectra without any well-defined emission lines from S-series and D-series. The device structure of Gr/hBN/Gr on top of a supporting hBN layer (Supplementary Figure 1c and 1d) shows emission lines of D-series while emission lines from S-series is absent or broadened dramatically.

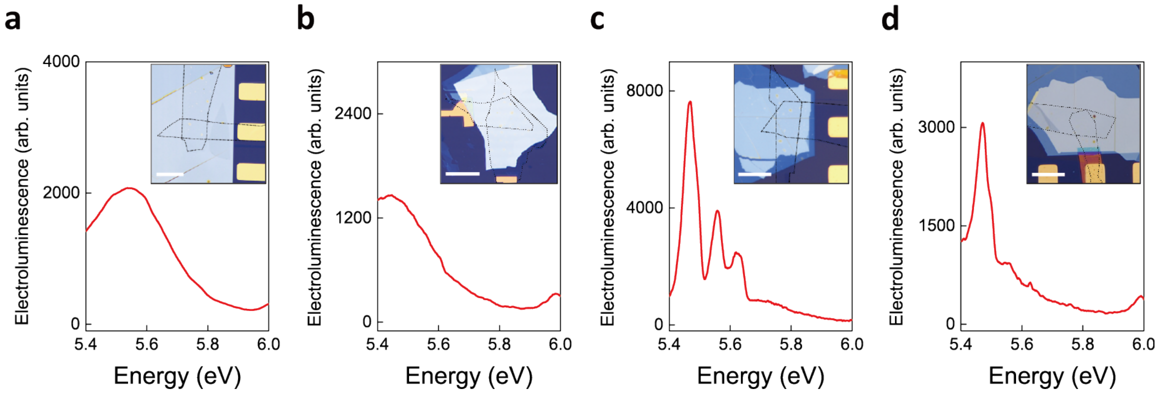

Supplementary Figure 1 Electroluminescence from the device structure without hBN encapsulation. a, **b**. Electroluminescence spectra from the Gr/hBN/Gr device alone. **c,d**. Electroluminescence spectra from the Gr/hBN/Gr device on top of a supporting layer of hBN. Insets show optical microscope images of the devices. Black dashed lines indicate graphene electrodes. The scale bar corresponds to 15 um.

On the other hand, electroluminescence spectra from the devices with hBN encapsulation (Supplementary Figure 2) show emission lines from both S-series and D-series. Insets of Supplementary Figure 2 show the device images and the thicknesses of the

emissive hBN layer. The devices exhibit electroluminescence EQE randomly scattered from 10^{-5} to 10^{-4} without any clear thickness dependence. Significantly improved quality of the electroluminescence spectra is possibly due to an atomically flat and clean interfaces and due to a protection from the external environment as demonstrated for atomically thin transition metal dichalcogenides[1, 2] and black phosphorous crystals[3]. Nevertheless, the detailed features in electroluminescence spectra vary from devices to devices even with the hBN encapsulation. For example, the devices in Supplementary Figure 2 show different intensity ratio for S-series and D-series. This can imply the devices possess different density of stacking faults inside the emissive layer or at the vdW interfaces which was created during the crystal growth or the device fabrication processes.

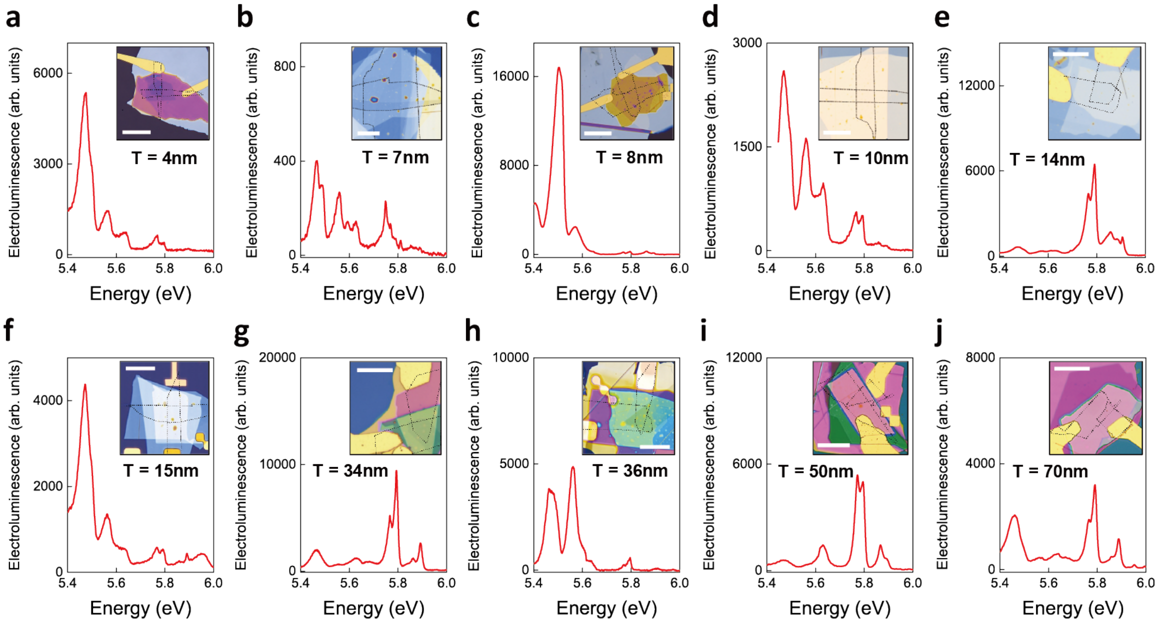

Supplementary Figure 2 Electroluminescence from the devices with hBN encapsulation. a-j. Electroluminescence spectra at a temperature of 10K from encapsulated vdW heterostructures of various emissive hBN layer thicknesses. **a**. 4 nm. **b**. 7 nm. **c**. 8 nm. **d**. 10 nm. **e**. 14 nm. **f**. 15 nm. **g**. 34 nm. **h**. 36 nm. **i**. 50 nm. **j**. 70 nm. Insets show optical microscope images of the devices for the electroluminescence measurement. Black dashed lines indicate graphene electrodes. The scale bar corresponds to 15 um. The hBN capping layers have the thickness range from 10 nm to 50 nm.

Supplementary Note 2. Photocurrent mapping at photoexcitation energy of 6.22 eV and 4.48 eV

Supplementary Figure 3b and 3c show scanning photocurrent images excited at photo-excitation energy at 6.22 eV (the above-gap excitation) and 4.48 eV (the below-gap excitation), respectively. The device is scanned under the same laser power of 2.5uW and the same bias voltage of 3 V. We observe nearly two orders of magnitude lower photocurrent (< 1.6 nA) for the below-gap excitation than the photocurrent (~ 60 nA) for the above-gap excitation. The weak photocurrent presumably arises from the optical transition from deep trap states[4] or the photo-assisted tunnelling process across the vertical interface of graphene electrodes and hBN[5].

Both the above- and the below-gap excitation show similar local spots (black dashed circles) where photocurrent is measured low in comparison to the neighbouring areas. In the same locations, we find bubbles at the vdW interfaces in optical microscope image (Supplementary Figure 3a). Therefore, this inhomogeneity can simply originate from inefficient carrier collection due to the vdW interface quality. On the other hand, the area marked with white dashed circle shows relatively weaker photocurrent only for the below-gap excitation. However, we do not find any notable feature in the optical image at the same location. This particular inhomogeneity is potentially due to inhomogeneous density of defects which contribute to generate photocurrent for the laser excitation at 4.48 eV. It will be interesting to measure photoluminescence spectra as a function of position to correlate the actual defect density and the photocurrent magnitude.

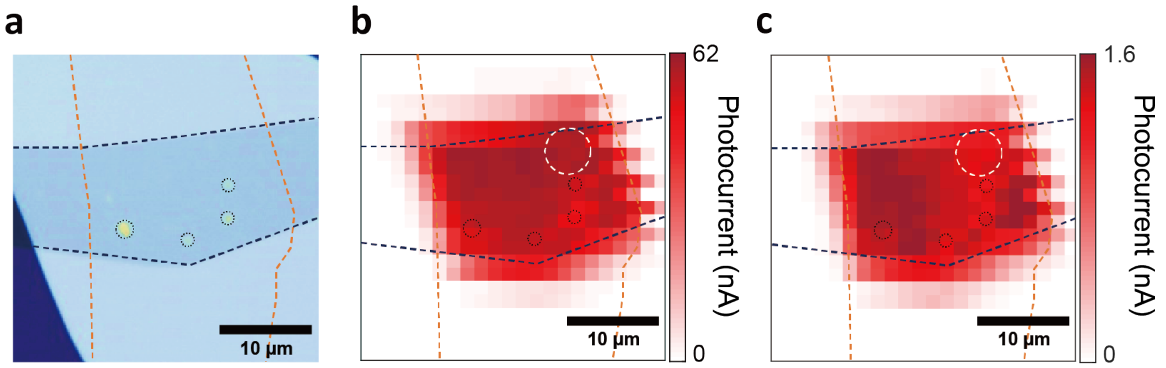

Supplementary Figure 3 Scanning photocurrent image. a. Optical microscope image of the device for photocurrent measurement. **b**. Scanning photocurrent image measured at photoexcitation energy of 6.22 eV. **c**. Scanning photocurrent image measured at photoexcitation energy of 4.48 eV. **b**. and **c**. are measured under the same bias voltage of $V_B = 3$ V. Orange and blue dashed lines indicate the top and the bottom graphene electrodes, respectively. Black dashed circles in **a**. indicate the interfacial bubbles in hBN vdW heterostructure. For photocurrent measurement, third and fourth harmonic generation of femtosecond laser pulses with 80 MHz repetition rate from Ti:Sapphire oscillator are utilized to generate the laser excitation at 4.48 eV and 6.22 eV, respectively.

Supplementary Note 3. I-V characteristics at a temperature of 77 K

I-V characteristics at a temperature of 77 K (red solid and dashed lines in Supplementary Figure 4) is similar to the characteristics at a temperature of 10 K, which is expected because Fowler-Nordheim tunneling current does not depend on the temperature. The I-V characteristics are replotted with respect to $\ln(I/{V_B}^2)$ and $1/|V_B|$ in Supplementary Figure 4b separately for the forward bias (blue solid line) and the reverse bias (blue dashed line). The excellent agreement with linear fitting (grey solid lines) indicates that Fowler-Nordheim tunneling is the major carrier conduction mechanism for both bias polarities. Nearly identical slopes indicate that tunneling current is dominated by a single type of charge carrier with the same effective mass and tunneling barrier height. According to the recent studies[6, 7], injection of holes is more efficient than electrons at the junction of graphene and hBN via Fowler-Nordheim tunneling, suggesting that holes are major charge carriers in our tunneling current. The offset between blue solid line and blue dashed line is possibly due to charged traps[8] and different effective tunneling areas of anodes (i.e. the electrodes for injecting holes) under the forward and reverse bias voltages.

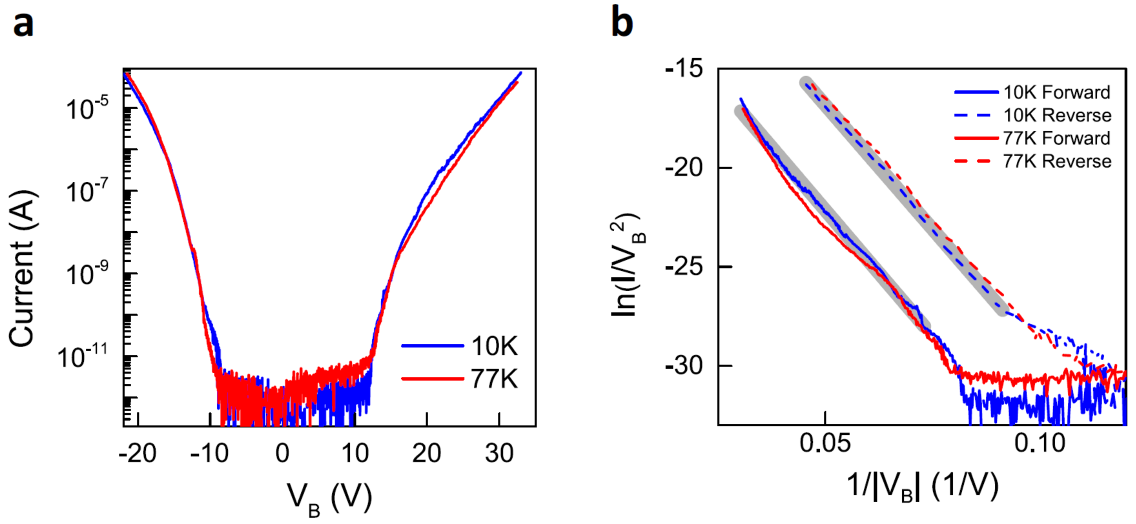

Supplementary Figure 4 I-V characterization via Fowler-Nordheim tunneling mechanism at 10 K and 77 K. a. I-V characteristics at 10 K and 77 K. **b**. I-V characteristics replotted with respect to $\ln(I/{V_B}^2)$ and $1/|V_B|$ for the forward bias (solid line) and the reverse bias (dashed line).

Supplementary Note 4. Observation of defect formation with I-V characteristics at room temperature

In order to understand I-V characteristics (Fig.3b in the main text) under low bias voltages at room temperature, we examine the effect of high electric fields on the emissive hBN layer. We have fabricated a device with the Gr/hBN/Gr structure with hBN thickness of 50 nm. First, we examine I-V characteristics under bias voltages within the range between 0 V and 20 V at room temperature (black solid line in Supplementary Figure 5a). No current is observed beyond the noise level, which is consistent with Fig.2d in the main text. However, once the bias voltage is applied over 53 V, we observe the sudden current increase. Afterwards, the same device exhibits significant current in I-V characteristics even under low bias voltages (red solid line in Supplementary Figure 5a), which reproduces qualitatively similar behaviour in Fig.3b of the main text. The I-V characteristic (red line in Supplementary Figure 5a) can be analyzed by the model based on Poole-Frenkel emission[9]. Poole-Frenkel emission originates from the emission of the thermally excited electrons from defect states into the conduction band of the dielectric under applied electric field. For Poole-Frenkel emission, the relationship between $\ln(I/V_B)$ and ${V_B}^{1/2}$ is linear. The I-V characteristics are replotted with respect to $\ln(I/V_B)$ and ${V_B}^{1/2}$ in Supplementary Figure 5b. We find that the linear fitting shows good agreement under the voltages above 4 V. Therefore, we demonstrate that the application of high electric field can form defects in the emissive hBN layer, which is captured by I-V characteristics under low bias voltages at room temperature. However, as shown in Fig.3 of the main text, the devices can still inject charge carriers via Fowler-Nordheim tunneling under high bias voltages without completely breaking the device, which provides electrons and holes for DUV electroluminescence.

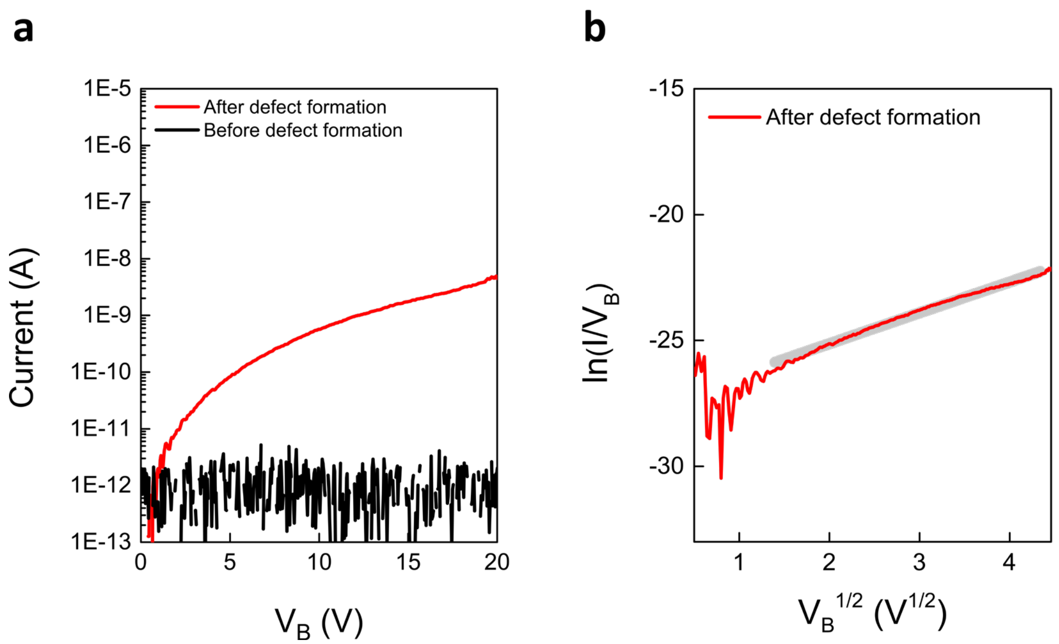

Supplementary Figure 5 I-V characterization via Poole-Frenkel emission mechanism at room temperature. **a**. I-V characteristics before defect formation (black solid line) and after defect formation (red solid line) at room temperature **b**. I-V characteristic of red solid line in (**a**) replotted with respect to $\ln(I/V_B)$ and $V_B{}^{1/2}$.

Supplementary Note 5. Measurement of external quantum efficiency for electroluminescence

The external quantum efficiency (EQE) is defined as the number of photons radiated toward the top side of the vdW heterostructures per the number of injected majority carriers *eN/I* (*N* is the number of radiated photons toward the top side per second, *e* is charge of electron, *I* is total current through hBN active layer between two graphene electrodes)[10]. According the recent study, injection of holes is much more efficient than electrons at the interface of graphene and hBN via Fowler-Nordheim tunnelling mechanism[6, 7]. Therefore, we assume one type of charge carrier is dominant in total current I in our devices.

In order to measure N, we measure the conversion coefficient C between N and integrated counts of spectrum per a minute in our home-built microscopy set-up. The conversion coefficient is defined as,

$$C = C_{setup} * T * \eta_{lens} \tag{1}$$

η_{lens} is the collection efficiency of our reflective objective[11]. Rays of electroluminescence within the critical angle (θ_c) defined by total internal reflection can escape from the van der Waals heterostructures. We assume the objective collects the conical part of these rays. We use a 15X reflective objective with numerical aperture of 0.28 and obscuration ratio of 27%. Due to the hBN encapsulation, the numerical aperture effectively decreases to *NA/n* (*NA* is numerical aperture of objective, *n* is refractive index of hBN at ~ 5.7 eV which is ~ 2.6[12]).

$$\eta_{\text{lens}} = 0.73 \cdot \frac{\int_0^{2\pi} d\varphi \cdot \int_0^{\sin^{-1}\left(\frac{NA}{n}\right)} \sin\theta d\theta}{\int_0^{2\pi} d\varphi \cdot \int_0^{\theta_c} \sin\theta d\theta} = 0.73 \cdot \frac{1-\cos(\sin^{-1}\frac{NA}{n})}{1-\cos\theta_c} = 0.055 \tag{2}$$

T is transmittance of CaF_2 window of cryostat in which hBN device is loaded.

$T = 0.87$

C_{setup} converts the number of photons collected by the objective to integrated counts of spectrum per a minute which is taking into account CCD quantum efficiency, grating efficiency, loss from mirror, lens, atmospheric absorption, and so on.

In order to measure C_{setup}, we use strongly attenuated DUV femtosecond laser at ~ 5.83 eV reflected from DUV mirror placed at the same location of the device.

Integrated counts of spectrum per minute is measured as 255.4 counts*eV/min per 2.36 pW (2530600 photons per second).

$$C_{\mathrm{setup}} = 255.4/2530600 = 1.0093*10^{-4} \qquad (3)$$

With this procedure, EQE is estimated to be EQE = $eN_{spectrum}/(CI)$ (N_{spectrum} is integrated counts of EL spectrum per a minute from 5.4 eV to 6 eV).

Supplementary Note 6. Spatial profiles of electroluminescence

The spatial profiles of DUV electroluminescence at room temperature are imaged by a CCD detector with a bandpass filter with full-width-half-maximum of ~ 1 eV centered at 5.8 eV. Supplementary Figure 6a-c are the optical microscope images of encapsulated electroluminescence devices. Supplementary Figure 6d-f are the spatial profiles under the forward bias. Supplementary Figure 6g-i are the spatial profiles under the reverse bias. The orange and blue dashed lines correspond to the top and the bottom electrodes, respectively. The electroluminescence signal is mostly concentrated in or near the area overlapping two graphene electrodes. The signal appears particularly strong at the corners or the edges of the area. This is due to enhancement of local electric field at the boundaries of electrodes, which facilitates carrier injection via Fowler-Nordheim tunneling and spreads charge carriers horizontally outside the overlap area[13].

For the device 2 and 3, interestingly, we find that electroluminescence signals appear dominantly on the edge of cathodes in the overlap area. This behaviour can be explained as following. According to the previous study[6, 7], the hole injection is more efficient than the electron injection at the interface of graphene and hBN due to smaller barrier height. As the bias voltage increase, initially only holes are injected along the edges of anodes where the electric field is enhanced. Electrons are not injected since higher electric field is required to inject electrons. Under higher bias voltages, holes are injected and spread through relatively wider area in the hBN emissive layer while electrons are mostly injected through the edges of cathodes. Therefore, holes and electrons can recombine and luminesce preferably appear along the edges of cathodes in the overlap area. This behavior can be clearly observed in the spatial profile at 10 K (Supplementary Figure 7).

However, as in the case of the device 1 (Supplementary Figure 6a) and device A at room temperature (the insets of Fig.3d and 3e of the main text), such behaviour is not observed for all the devices. The discrepancy can be potentially explained by defects which can alter the carrier flow or trap carriers depending on the bias polarities and temperature.

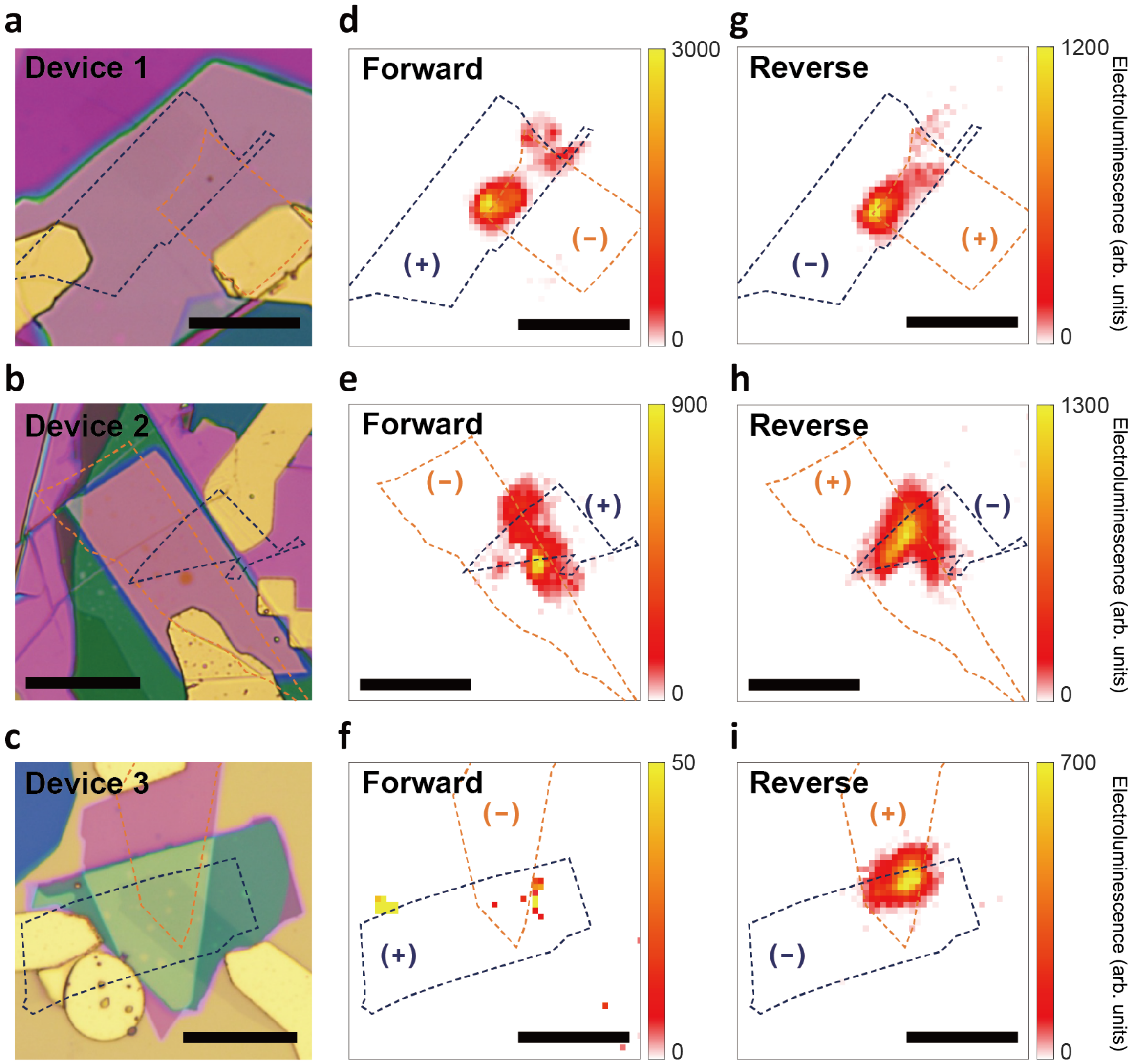

Supplementary Figure 6 Optical microscope images and spatial profiles of DUV electroluminescence at room temperature. a-c. Optical microscope images of the hBN encapsulated devices (scale bar 10μm). **d-f.** Spatial profiles of DUV electroluminescence under the forward bias. **g-i.** Spatial profiles of DUV electroluminescence under the reverse bias. Orange and blue dashed lines mark the top and the bottom graphene electrodes, respectively. (+) and (-) indicate anodic and cathodic graphene electrodes, respectively. The spatial profiles of DUV electroluminescence at room temperature are imaged by a CCD detector with a bandpass filter with full-width-half-maximum of ~ 1 eV centered at 5.8 eV.

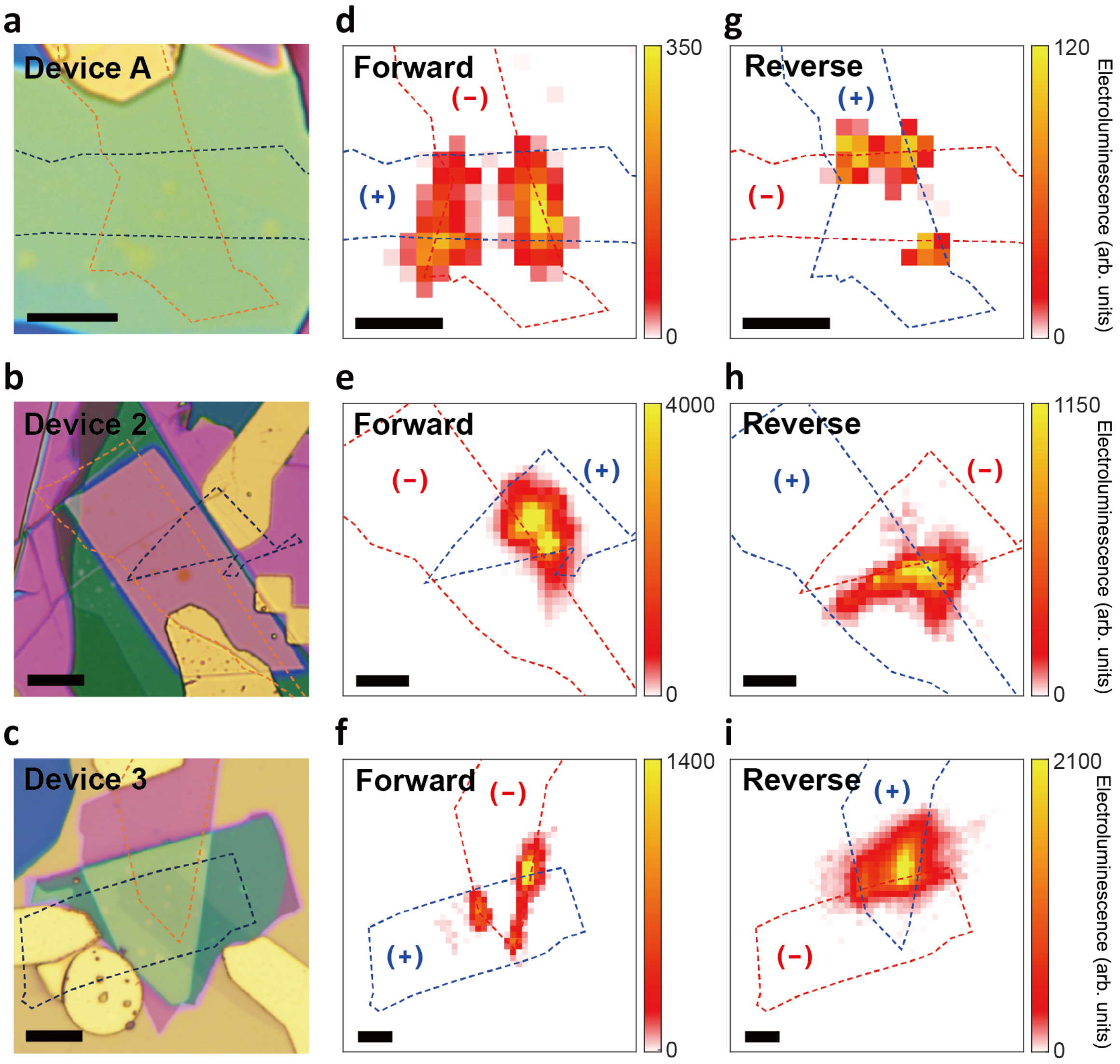

Supplementary Figure 7 Optical microscope images and spatial profiles of DUV electroluminescence at 10 K. a-c. Optical microscope images of the hBN encapsulated devices (scale bar 5μm). Orange and blue dashed lines mark the top and the bottom graphene electrodes, respectively. **d-f.** Spatial profiles of DUV electroluminescence under the forward bias. **g-i.** Spatial profiles of DUV electroluminescence under the reverse bias. Red and blue dashed lines mark the cathode and the anode, respectively. The spatial profiles of DUV electroluminescence at 10 K are imaged by a CCD detector with a bandpass filter with full-width-half-maximum of ~ 1 eV centered at 5.8 eV.

Supplementary Note 7. Vertical linecuts of PLE spectra for S_{1-4} and $D_{1-2,4}$ lines

Supplementary Figure 8 shows vertical linecuts of Fig.4c along S- and D-lines of photoluminescence spectrum. Luminescence intensity of S_{1-4} lines increases with the photoexcitation above 6 eV and shows the excitation resonance at 6.07 eV. Luminescence intensity of $D_{1-2,4}$ lines, on the other hand, shows additional excitation resonance at 5.97 eV. The artifacts from the elastic scattering residue of excitation laser (purple dashed line in Fig.4c) are marked by black arrows at Supplementary Figure 8e and 8f.

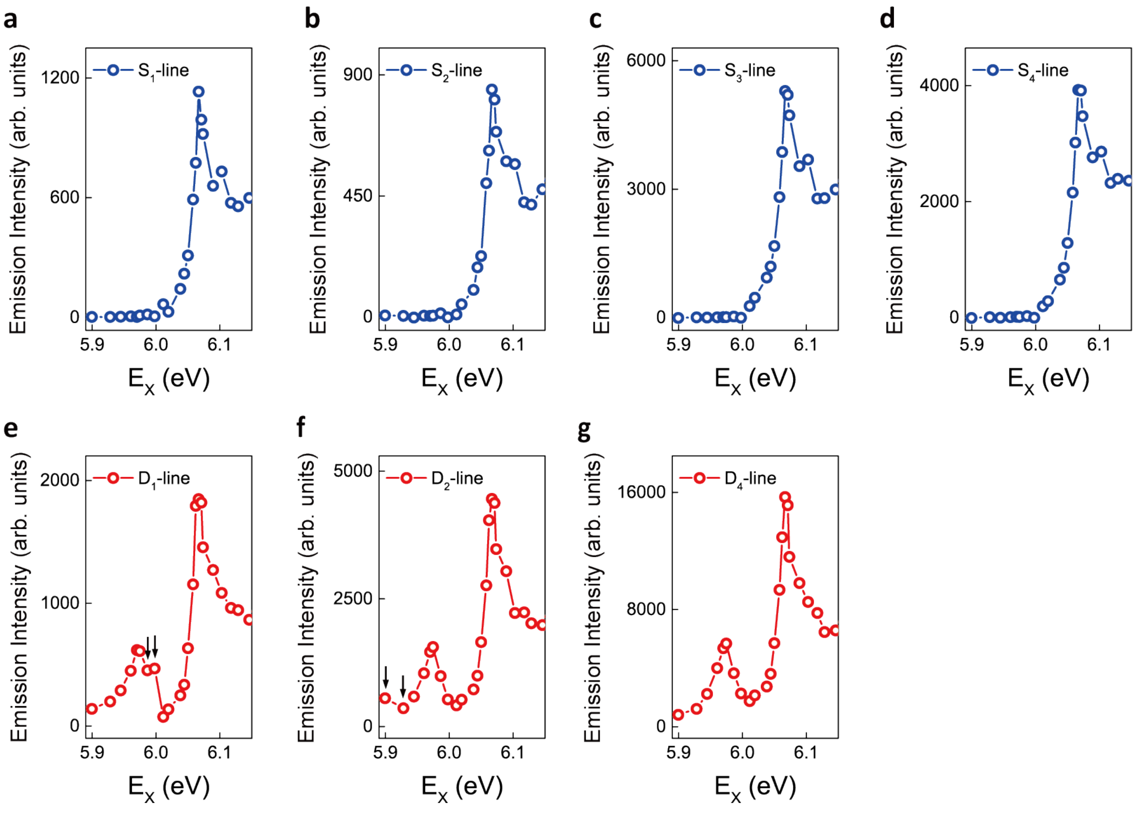

Supplementary Figure 8 Vertical linecuts of PLE color map for S_{1-4} and $D_{1-2,4}$ lines. a-d. Excitation spectra of S-series photoluminescence intensity. **e-g.** Excitation spectra of D-series photoluminescence intensity. Black arrows indicate artifact data points from excitation laser residue.

Supplementary References

1 Mak, K. F. & Shan, J. Photonics and optoelectronics of 2D semiconductor transition metal dichalcogenides. *Nature Photonics* **10**, 216-226, doi:10.1038/nphoton.2015.282 (2016).

2 Rhodes, D., Chae, S. H., Ribeiro-Palau, R. & Hone, J. Disorder in van der Waals heterostructures of 2D materials. *Nature Materials* **18**, 541-549, doi:10.1038/s41563-019-0366-8 (2019).

3 Yoon, S. *et al.* Electrical control of anisotropic and tightly bound excitons in bilayer phosphorene. *Physical Review B* **103**, L041407, doi:10.1103/PhysRevB.103.L041407 (2021).

4 Remes, Z., Nesladek, M., Haenen, K., Watanabe, K. & Taniguchi, T. The optical absorption and photoconductivity spectra of hexagonal boron nitride single crystals. *Phys. Stat. Sol. (a)* **202**, 2229-2233, doi:10.1002/pssa.200561902 (2005).

5 Yadav, D. *et al.* Terahertz wave generation and detection in double-graphene layered van der Waals heterostructures. *2D Materials* **3**, 045009, doi:10.1088/2053-1583/3/4/045009 (2016).

6 Hattori, Y., Taniguchi, T., Watanabe, K. & Nagashio, K. Determination of Carrier Polarity in Fowler–Nordheim Tunneling and Evidence of Fermi Level Pinning at the Hexagonal Boron Nitride/Metal Interface. *ACS Applied Materials & Interfaces* **10**, 11732-11738, doi:10.1021/acsami.7b18454 (2018).

7 Lee, J.-H. *et al.* Semiconductor-less vertical transistor with I_{ON}/I_{OFF} of 10^6. *Nature Communications* **12**, 1000, doi:10.1038/s41467-021-21138-y (2021).

8 Hattori, Y., Taniguchi, T., Watanabe, K. & Nagashio, K. Impact ionization and transport

properties of hexagonal boron nitride in a constact-voltage measurement, *Physical Review B* **97**, 045425, doi.org/10.1103/PhysRevB.97.045425 (2018)

9 Chiu, F.-C. A Review on Conduction Mechanisms in Dielectric Films. *Advances in Materials Science and Engineering* **2014**, 578168, doi:10.1155/2014/578168 (2014).

10 Kasap, S. O. Optoelectronics and Photonics: Principles and Practices. *Pearson* (2013).

11 Withers, F. *et al.* Light-emitting diodes by band-structure engineering in van der Waals heterostructures. *Nature Materials* **14**, 301-306, doi:10.1038/nmat4205 (2015).

12 Stenzel, O. *et al.* The Optical Constants of Cubic and Hexagonal Boron Nitride Thin Films and Their Relation to the Bulk Optical Constants. *phys. Stat. Sol. (a)* **158**, 281, doi:10.1002/pssa.2211580130 (1996).

13 Schubert, E. F. *Light-Emitting Diodes*. 2 edn, (Cambridge University Press, 2006).
